# Development and Preliminary Validation of a Knowledge, Attitude, and Practice Questionnaire Assessing Parental Self-Healthcare for Respiratory Tract Infections in Children With Cerebral Palsy in Malaysia

**DOI:** 10.7759/cureus.103317

**Published:** 2026-02-09

**Authors:** Riham M. K Abualeinein, Sazlina Kamaralzaman, Nur Zakiah Mohd Saat

**Affiliations:** 1 Center for Rehabilitation and Special Needs Studies (iCaRehab), Faculty of Health Sciences, Universiti Kebangsaan Malaysia, Kuala Lumpur, MYS; 2 Programme of Biomedical Sciences, Centre of Community Health Studies (ReaCH), Faculty of Health Sciences, Universiti Kebangsaan Malaysia, Kuala Lumpur, MYS

**Keywords:** attitude, cerebral palsy, content validity index, internal consistency reliability, knowledge, parents, practice, questionnaire development, respiratory tract infections

## Abstract

Background

Children with cerebral palsy (CP) are vulnerable to recurrent respiratory tract infections (RTIs), contributing to morbidity and repeated hospital admissions. Parents and primary caregivers play a central role in prevention, early recognition, and home management; however, Malaysia‑specific validated tools to assess parental knowledge, attitudes, and practices (KAP) regarding RTIs in CP are limited. Developing and preliminarily validating a KAP questionnaire is essential for assessing parental self-healthcare related to RTIs among children with CP in Malaysia.

Methodology

A methodological study was conducted to develop and preliminarily validate a KAP questionnaire assessing parental self-healthcare related to RTIs among children with CP. Questionnaire items were developed based on a literature review and the KAP framework. Content validity was assessed by a panel of six experts using the Content Validity Index (CVI). Pilot testing was conducted among 33 parents of children with neurodevelopmental disabilities to evaluate item clarity, feasibility, and internal consistency reliability. Reliability analysis was performed using IBM SPSS Statistics for Windows, Version 23 (Released 2016; IBM Corp., Armonk, New York, United States), while CVI calculations were conducted using Microsoft Excel (Microsoft Corp., Redmond, WA, USA).

Results

Item-level content validity indices ranged from 0.67 to 1.00. All domains demonstrated excellent scale-level content validity (S-CVI/Ave ≥ 0.90). Minor wording revisions were made based on expert feedback, with no item removal. Internal consistency reliability was acceptable to good across domains (knowledge α = 0.82; attitude α = 0.739; practice α = 0.795) and excellent for the overall scale (α = 0.905).

Conclusion

The developed questionnaire demonstrated excellent content validity and satisfactory internal consistency reliability, supporting its preliminary psychometric adequacy. Further validation among parents of children with CP is recommended to assess construct validity, test-retest reliability, and broader applicability.

## Introduction

Children with cerebral palsy (CP) have complex neuromuscular and systemic comorbidities that require long-term caregiver involvement [1]. Respiratory illness represents a leading cause of morbidity, emergency department attendance, and hospitalization among children and young people with CP, particularly among those with more severe motor impairment [1,2]. This increased vulnerability is attributed to multifactorial mechanisms, including impaired airway clearance, ineffective cough, dysphagia-related aspiration, and recurrent chest infections [1,2].

International consensus recommendations emphasize the importance of early identification of respiratory risk factors, caregiver education, and preventive strategies to reduce recurrent respiratory tract infections (RTIs) and associated healthcare burden among individuals with CP [1]. Despite advances in clinical management, respiratory complications remain a major contributor to preventable hospital admissions and long-term morbidity, underscoring the critical role of caregivers in daily respiratory risk reduction and early intervention [2,3].

Parents and primary caregivers play a central role in the prevention and home management of RTIs among children with CP. Their responsibilities include recognizing early respiratory symptoms, implementing preventive measures, ensuring safe feeding and positioning practices, seeking timely medical care, and adhering to healthcare recommendations. However, existing evidence indicates substantial variability in caregiver knowledge and home-based practices related to respiratory health in children with CP, which may contribute to delayed healthcare-seeking behavior and avoidable complications [4,5].

The knowledge, attitude, and practice (KAP) framework provides a structured approach to examining caregiver-related behaviors by assessing what caregivers know, how they perceive and prioritize health risks, and how they translate knowledge into daily caregiving practices. KAP-based instruments have been widely used in public health research to identify behavioral gaps, guide targeted educational interventions, and evaluate caregiver preparedness across diverse health conditions and caregiving contexts [6-8]. Despite the recognized importance of caregiver engagement in respiratory care, validated and context-specific instruments assessing parental self-healthcare related to RTIs among children with CP remain limited in Malaysia.

In the Malaysian context, parents and caregivers of children with CP face substantial caregiving, financial, and healthcare-related burdens, which may further influence their capacity to effectively manage recurrent respiratory health problems at home [9]. This underscores the need for culturally appropriate and context-specific assessment tools to support systematic caregiver evaluation and inform targeted interventions within the local healthcare setting.

To address this gap, the present study aimed to develop and preliminarily validate a KAP questionnaire designed to assess parental self-healthcare related to RTIs among children with CP in Malaysia. The specific objectives were to evaluate the content validity of the questionnaire through expert review and to assess its preliminary internal consistency reliability through pilot testing.

## Materials and methods

Study design

This study employed a descriptive methodological design conducted between November 2024 and February 2025 to develop and preliminarily validate a KAP questionnaire assessing parental self-healthcare related to RTIs among children with CP in Malaysia. The validation process comprised two stages: content validity and pilot testing for preliminary internal consistency reliability, in line with recommended best practices for early-stage questionnaire development [10].

Study setting

The study was conducted through non-governmental organizations (NGOs) that provide support services for children with disabilities across Malaysia.

Sample size and data collection

Data collection commenced only after formal written approval was obtained from the participating institutions. The questionnaire was distributed to eligible parents via a Google Forms link (Google LLC, Mountain View, CA, USA) by the administrative staff of the approved organizations through official communication channels.

Participants for the pilot testing phase were recruited using a convenience sampling approach through participating NGOs. The pilot sample size was determined in accordance with methodological recommendations, indicating that approximately 30 participants are adequate for preliminary reliability assessment in pilot studies [11]. A total of 35 Malaysian parents of children under 18 years of age with neurodevelopmental disabilities were initially recruited. Eligible participants were required to be able to complete the questionnaire independently. Participants with incomplete questionnaire responses were excluded. Following data screening, three participants were excluded, resulting in a final pilot sample of 33 parents.

Parents of children with Down syndrome were included alongside those caring for children with CP during the pilot phase, as the primary objective at this stage was to assess instrument performance rather than condition-specific outcomes. Children with Down syndrome experience a high burden of RTIs and require continuous caregiver involvement in respiratory monitoring and preventive care, which overlaps with caregiving demands observed in CP [12,13].

Study tool

The questionnaire was developed by the research team based on a review of relevant literature and previously published KAP studies involving caregivers of children with CP [4,5], and guided by established principles for scale development and validation in health research [10].

The initial version of the questionnaire was drafted in English and subsequently translated into the Malay language by bilingual professionals. Both the English and Malay versions were evaluated during the content validity process. Based on expert feedback, minor wording and phrasing revisions were made to improve clarity and readability. No items were removed, as comments related primarily to wording rather than item relevance. Following these revisions, the finalized version was used during the pilot reliability phase.

Content validity was assessed by a panel of six experts, including physicians involved in the clinical management of children with CP and academic lecturers with at least five years of experience in KAP questionnaire development. Experts evaluated each item based on relevance, clarity, simplicity, and ambiguity using a four-point rating scale ranging from 1 (not relevant) to 4 (very relevant). Item-level content validity index (I-CVI) and scale-level content validity index/average (S-CVI/Ave) values were calculated based on expert agreement, in accordance with established guidelines [14-16].

The final questionnaire comprised three domains: knowledge (six items), attitude (seven items), and practice (eight items). Responses to all items were recorded using a five-point Likert-type scale (1 = strongly agree, 2 = agree, 3 = disagree, 4 = strongly disagree, and 5 = not sure). The full questionnaire is provided in the Appendices.

Data analysis

Data analysis was conducted using Microsoft Excel 2016 (Microsoft Corporation, Redmond, WA, USA) for content validity index calculations and IBM SPSS Statistics for Windows, Version 23 (Released 2016; IBM Corp., Armonk, New York, United States) for statistical analysis. Descriptive statistics were used to summarize the demographic characteristics of parents and expert validators.

I-CVI value of ≥ 0.78 was considered acceptable for six experts, while an S-CVI/Ave value of ≥ 0.90 indicated excellent content validity [15,16]. Internal consistency reliability was assessed using Cronbach’s alpha, with values of ≥ 0.70 considered acceptable for preliminary reliability assessment. Corrected item-total correlation values exceeding 0.30 were considered indicative of acceptable item discrimination, in line with the COnsensus-based Standards for the selection of health Measurement INstruments (COSMIN) recommendations [17] and established content validation guidelines [18].

Ethical considerations

Ethical approval was obtained from the Universiti Kebangsaan Malaysia Research Ethics Committee (JEP-2023-919). Written informed consent was obtained from all participants prior to participation. Participant confidentiality and voluntary participation were ensured throughout the study.

## Results

Expert panel characteristics

Six experts participated in the content validity assessment of the questionnaire. The majority were female (83.3%) and aged between 40 and 49 years (50.0%). All experts held doctoral-level qualifications and had more than 10 years of professional experience in relevant fields. The panel represented diverse disciplines, including biomedical sciences, public health, occupational therapy, and physiotherapy (Table 1).

**Table 1:** Demographic characteristics of the expert panel

Variable	Frequency (N)	Percent (%)
Gender		
Female	5	83.3
Male	1	16.7
Age Group (Years)		
30-39	2	33.3
40-49	3	50
50-59	1	16.7
Years of Experience		
10-14	2	33.3
15-19	3	50
25-29	1	16.7
Highest Qualification		
PhD	6	100
Specialization		
Biomedical Science	3	50
Public Health	1	16.7
Occupational Therapy	1	16.7
Physiotherapy	1	16.6

Content validity

The content validity assessment demonstrated strong agreement among experts across all questionnaire domains. I-CVI values ranged from 0.67 to 1.00 for items within the KAP domains, with most items meeting or exceeding the recommended threshold of 0.78.

The S-CVI/Ave was 0.92 for the knowledge domain, 0.995 for the attitude domain, and 0.97 for the practice domain, exceeding the recommended criterion of 0.90. Items with I-CVI values below the recommended threshold were revised to improve wording clarity and contextual relevance based on expert feedback (Tables 2-4).

**Table 2:** Item-level content validity indices for the knowledge domain I-CVI: item-level content validity index; S-CVI/Ave: scale-level content validity index/average

Item	Relevance (I-CVI)	Clarity (I-CVI)	Simplicity (I-CVI)	Ambiguity (I-CVI)	S-CVI/Ave
Knowledge item 1	0.83	1	1	1	0.96
Knowledge item 2	0.83	1	1	1	0.96
Knowledge item 3	0.83	1	1	1	0.96
Knowledge item 4	0.83	1	1	1	0.96
Knowledge item 5	0.67	0.83	1	0.83	0.83
Knowledge item 6	0.67	0.83	0.83	0.83	0.79
Overall (S-CVI/Ave)	0.78	0.97	0.97	0.94	0.92

**Table 3:** Item-level content validity indices for the attitude domain I-CVI: item-level content validity index; S-CVI/Ave: scale-level content validity index/average

Item	Relevance (I-CVI)	Clarity (I-CVI)	Simplicity (I-CVI)	Ambiguity (I-CVI)	S-CVI/Ave
Attitude item 1	1	1	1	1	1
Attitude item 2	1	1	1	1	1
Attitude item 3	1	1	1	0.83	0.96
Attitude item 4 to 7	1	1	1	1	1
Overall (S-CVI/Ave)	1	1	1	0.98	0.995

**Table 4:** Item-level content validity indices for the practice domain I-CVI: item-level content validity index; S-CVI/Ave: scale-level content validity index/average

Item	Relevance (I-CVI)	Clarity (I-CVI)	Simplicity (I-CVI)	Ambiguity (I-CVI)	S-CVI/Ave
Practice item 1	1	1	1	1	1
Practice item 2	1	1	1	1	1
Practice item 3	0.83	1	0.83	1	0.92
Practice item 4	1	1	1	1	1
Practice item 5	1	1	1	1	1
Practice item 6	1	1	1	1	1
Practice item 7	0.67	1	1	1	0.92
Practice item 8	0.67	1	1	1	0.92
Overall (S-CVI/Ave)	0.9	1	0.98	1	0.97

Pilot participant characteristics

Of the 35 parents initially recruited for pilot testing, three were excluded due to incomplete questionnaire responses. The final sample comprised 33 parents of children with neurodevelopmental disabilities, including parents of children with CP (n = 9) and parents of children with Down syndrome (n = 24). All respondents were female, with half aged between 30 and 39 years. Most participants had completed at least a bachelor’s degree, and the majority were employed in the private sector (Table 5).

**Table 5:** Demographic characteristics of pilot participants

Variable	Frequency (N)	Percent (%)
Age (Years)		
<30	7	21.2
30-39	17	51.5
40-49	3	9.1
≥50	6	18.2
Gender		
Female	33	100
Education Level		
Secondary School	6	18.2
Diploma	6	18.2
Bachelor’s Degree	10	30.3
Postgraduate	7	21.2
Others	4	12.1
Occupation		
Private Sector Employee	23	69.7
Self-Employed/Business Owner	4	12.1
Housewife	3	9.1
Others	3	9.1

Internal consistency reliability

The questionnaire demonstrated acceptable to good internal consistency reliability across all domains. Cronbach’s alpha coefficients were 0.82 for the knowledge domain, 0.739 for the attitude domain, and 0.795 for the practice domain. The overall scale demonstrated excellent internal consistency, with a Cronbach’s alpha of 0.905 (Table 6).

**Table 6:** Internal consistency reliability by domain

Domain	Number of Items	Cronbach’s Alpha (α)
Knowledge	6	0.82
Attitude	7	0.739
Practice	8	0.795
Overall Scale	21	0.905

Corrected item-total correlation values exceeded 0.30 for most items across domains, indicating adequate item contribution. Deletion of individual items did not result in meaningful improvements in Cronbach’s alpha values, supporting retention of all items at this stage (Tables 7-9).

**Table 7:** Internal consistency reliability of the knowledge domain (n = 33) Note: Full item wording is provided in the Appendices.

Item	Corrected Item-Total Correlation	Cronbach’s Alpha if Item Deleted
Knowledge item 1	0.523	0.802
Knowledge item 2	0.685	0.776
Knowledge item 3	0.825	0.743
Knowledge item 4	0.715	0.758
Knowledge item 5	0.721	0.759
Knowledge item 6	0.082	0.863
Overall Cronbach’s alpha (α)		0.82

**Table 8:** Internal consistency reliability of the attitude domain (n = 33) Note: Full item wording is provided in the Appendices.

Item	Corrected Item-Total Correlation	Cronbach’s Alpha if Item Deleted
Attitude item 1	0.412	0.718
Attitude item 2	0.664	0.649
Attitude item 3	0.611	0.669
Attitude item 4	0.318	0.738
Attitude item 5	0.398	0.722
Attitude item 6	0.358	0.734
Attitude item 7	0.454	0.708
Overall Cronbach’s alpha (α)		0.739

**Table 9:** Internal consistency reliability of the practice domain (n = 33) Note: Full item wording is provided in the Appendices.

Item	Corrected Item-Total Correlation	Cronbach’s Alpha if Item Deleted
Practice item 1	0.618	0.767
Practice item 2	0.524	0.773
Practice item 3	0.398	0.787
Practice item 4	0.612	0.759
Practice item 5	0.455	0.785
Practice item 6	0.667	0.742
Practice item 7	0.475	0.781
Practice item 8	0.575	0.772
Overall Cronbach’s alpha (α)		0.795

## Discussion

This study developed and preliminarily validated a KAP questionnaire to assess parental self-healthcare related to RTIs among children with CP in Malaysia. The findings demonstrated excellent content validity and acceptable to good internal consistency reliability across the questionnaire domains, supporting the preliminary psychometric adequacy of the instrument. These results align with established methodological frameworks for early-stage questionnaire development, which emphasize the importance of confirming content validity and internal consistency before proceeding to large-scale construct validation and factor analysis [9,10].

A key strength of the present study lies in the robust content validity demonstrated across all questionnaire domains. The high scale-level content validity indices (S-CVI/Ave ≥ 0.90) obtained for the KAP domains indicate strong agreement among expert reviewers regarding item relevance, clarity, and representativeness. Importantly, these results suggest that the questionnaire items were not only theoretically appropriate but also practically interpretable and contextually relevant for assessing parental self-healthcare behaviors related to RTIs within the Malaysian caregiving environment. Such high levels of content validity are particularly important during early instrument development, as they provide confidence that the tool adequately captures the intended construct prior to further psychometric testing [14-17].

Although a small number of items demonstrated comparatively lower I-CVI values, these findings were addressed through targeted wording refinement rather than item elimination. This approach reflects best practices in scale development, where content validity assessment is viewed as an iterative process aimed at improving item clarity while preserving conceptual breadth and coverage [10,14]. Retaining these items ensured that important aspects of parental self-healthcare behaviors were not prematurely excluded, particularly in a newly developed instrument intended for use in a complex caregiving context such as CP.

Pilot testing further demonstrated acceptable to good internal consistency reliability across all domains, with Cronbach’s alpha values ranging from 0.739 to 0.82 and excellent reliability observed for the overall scale (α = 0.905). Reliability coefficients within this range are considered appropriate for pilot studies and early phases of instrument development [18,19]. These findings indicate adequate interrelatedness among items within each domain and suggest that the questionnaire consistently measures related aspects of parental KAPs. Importantly, reliability values within this range are considered appropriate for pilot studies and early phases of instrument development, where the primary objective is to establish preliminary measurement stability rather than definitive internal structure [18].

Item-level reliability analysis provided additional support for the robustness of the questionnaire. Corrected item-total correlation values exceeded the recommended threshold for most items, and the removal of individual items did not result in meaningful improvements in Cronbach’s alpha values. This finding indicates that each item contributes uniquely to its respective domain and supports the decision to retain the full set of items for subsequent validation phases. Preserving all items at this stage is methodologically sound, as it allows for more comprehensive evaluation of construct validity and factor structure in future studies involving larger and more representative samples [10,18].

The inclusion of parents of children with Down syndrome alongside parents of children with CP during the pilot phase warrants consideration. This decision was methodologically justified, as the primary aim of pilot testing was to evaluate item clarity, feasibility, and preliminary reliability rather than condition-specific outcomes. Children with Down syndrome experience a high burden of RTIs and require continuous caregiver involvement in respiratory monitoring and preventive care, which closely parallels caregiving demands observed in CP [12,13]. Limiting this heterogeneous sample to the pilot phase did not compromise the intended application of the questionnaire, which remains specifically designed for parents of children with CP and will be further validated within this target population in subsequent research.

From a public health perspective, KAP-based instruments are widely used to identify caregiver knowledge gaps, inform health education strategies, and guide prevention-oriented interventions across diverse healthcare settings [7,8]. In the context of CP, caregiver knowledge and self-care practices play a critical role in the early recognition and management of RTIs, which remain a leading cause of morbidity and hospital admission. Evidence from Malaysia and other settings suggests that caregiver perceptions and self-care behaviors significantly influence health outcomes among children with disabilities [20]. Moreover, structured educational interventions targeting caregivers of children with CP have been shown to improve caregiver knowledge and home-based respiratory care practices, leading to better management of respiratory complications and improved child health outcomes [21,22].

In addition to caregiver education, strengthening family-professional collaboration has been identified as a key factor in improving functional outcomes for children with CP while reducing caregiver burden and enhancing quality of life [23]. The availability of a valid and reliable tool to assess parental self-healthcare behaviors, therefore, provides an important foundation for both clinical practice and community-based interventions, enabling healthcare professionals to systematically identify caregiver needs and tailor support strategies accordingly.

Within the Malaysian context, parents and caregivers of children with CP face substantial caregiving, financial, and healthcare-related burdens that may limit their capacity to consistently implement effective home-based respiratory care [9]. The strong content validity and satisfactory internal consistency reliability observed in this study suggest that the developed questionnaire is well-aligned with the lived experiences and caregiving responsibilities of Malaysian parents. This alignment enhances the potential utility of the instrument for identifying parental educational needs, informing culturally appropriate interventions, and supporting broader health system planning aimed at reducing preventable respiratory complications among children with CP.

Overall, the findings of this study indicate that the developed KAP questionnaire demonstrates sound preliminary psychometric properties and is suitable for early-stage application. Future studies involving larger samples of parents of children with CP are warranted to further evaluate construct validity, test-retest reliability, and responsiveness to intervention, thereby strengthening the evidence base for its use in both research and clinical settings.

Limitations

This study has several limitations that should be acknowledged. Psychometric evaluation was limited to content validity and preliminary internal consistency reliability, while other properties, such as construct validity and test-retest reliability, were not assessed, as the primary aim was early-stage instrument development. Additionally, pilot testing was conducted using a relatively small and heterogeneous sample, which was appropriate for evaluating item clarity and feasibility but limits generalizability. In addition, all participants in the pilot phase were female, which may further limit generalizability. Parents of children with Down syndrome were included solely during the pilot testing phase to assess item clarity, feasibility, and preliminary internal consistency reliability rather than condition-specific outcomes. This heterogeneity was confined to the preliminary phase. Future studies involving larger and more representative samples of parents of children with CP are needed to further validate the questionnaire and confirm its broader applicability.

## Conclusions

This study developed and preliminarily validated a KAP questionnaire to assess parental self-healthcare related to RTIs among children with CP in Malaysia. The instrument demonstrated excellent content validity and satisfactory internal consistency reliability, supporting its suitability for early-stage application. This questionnaire may serve as a useful tool for assessing caregiver preparedness, guiding targeted educational interventions, and informing prevention-oriented strategies within clinical and community care settings. Further validation studies are recommended to evaluate construct validity, test-retest reliability, and broader applicability among parents of children with CP.

## Appendices

Knowledge domain

1.      Saya sedar tentang masalah pernafasan yang biasa dihadapi oleh kanak-kanak yang mengalami palsi serebral. (*I am aware of the typical respiratory issues that children with cerebral palsy may have*.)

☐  Sangat setuju (Strongly agree) ☐  Setuju (Agree) ☐  Tidak setuju (Disagree) ☐  Sangat tidak setuju (Strongly disagree) ☐  Tidak pasti (Not sure)

2.      Saya sedar bagaimana kesihatan umum anak saya mungkin terjejas oleh jangkitan pernafasan (*I am aware of how my child's general health may be affected by respiratory infections*.)

☐  Sangat setuju (Strongly agree) ☐  Setuju (Agree) ☐  Tidak setuju (Disagree) ☐  Sangat tidak setuju (Strongly disagree) ☐  Tidak pasti (Not sure)

3.      Saya sedar akan tanda-tanda dan gejala-gejala yang jelas bagi jangkitan pernafasan yang dialami oleh kanak-kanak. (*I am aware of the telltale signs and symptoms of respiratory infections in children*.)

☐  Sangat setuju (Strongly agree) ☐  Setuju (Agree) ☐  Tidak setuju (Disagree) ☐  Sangat tidak setuju (Strongly disagree) ☐  Tidak pasti (Not sure)

4.      Saya sedar kanak-kanak yang mengalami palsi serebral perlu diperiksa untuk masalah pernafasan lebih kerap daripada kanak-kanak dengan perkembangan tipikal. (*I know children with cerebral palsy need to be checked for respiratory problems more often than children with typical development*.)

☐  Sangat setuju (Strongly agree) ☐  Setuju (Agree) ☐  Tidak setuju (Disagree) ☐  Sangat tidak setuju (Strongly disagree) ☐  Tidak pasti (Not sure)

5.      Ahli profesional kesihatan telah memberikan saya pengetahuan yang cukup tentang kesihatan pernafasan kanak-kanak yang mengalami palsi serebral. (*Healthcare professionals have given me enough knowledge on the respiratory health of children with cerebral palsy*.)

☐  Sangat setuju (Strongly agree) ☐  Setuju (Agree) ☐  Tidak setuju (Disagree) ☐  Sangat tidak setuju (Strongly disagree) ☐  Tidak pasti (Not sure)

6.      Saya sedar bahawa saya tidak tahu cukup tentang pengurusan kesihatan pernafasan untuk menjaga anak saya dengan betul.(*I am aware that I do not have sufficient knowledge about respiratory health management to properly care for my child*.)

☐  Sangat setuju (Strongly agree) ☐  Setuju (Agree) ☐  Tidak setuju (Disagree) ☐  Sangat tidak setuju (Strongly disagree) ☐  Tidak pasti (Not sure)

Attitude domain

1.      Saya berpendapat bahawa penyakit pernafasan anak saya menimbulkan risiko kesihatan yang besar. (*I *think my child's respiratory illnesses pose a major health risk.)

☐  Sangat setuju (Strongly agree) ☐  Setuju (Agree) ☐  Tidak setuju (Disagree) ☐  Sangat tidak setuju (Strongly disagree) ☐  Tidak pasti (Not sure)

2.      Saya yakin dengan kemampuan saya untuk mengurus kesihatan pernafasan anak saya. (*I feel confident in my ability to manage my child's respiratory health.*)

☐  Sangat setuju (Strongly agree) ☐  Setuju (Agree) ☐  Tidak setuju (Disagree) ☐  Sangat tidak setuju (Strongly disagree) ☐  Tidak pasti (Not sure)

3.      Saya percaya bahawa dengan rawatan dan penjagaan yang betul, penyakit pernafasan boleh dielakkan dengan jayanya. (*I believe that with the right treatment and care, respiratory illnesses may be successfully avoided*.)

☐  Sangat setuju (Strongly agree) ☐  Setuju (Agree) ☐  Tidak setuju (Disagree) ☐  Sangat tidak setuju (Strongly disagree) ☐  Tidak pasti (Not sure)

4.      Saya berpendapat adalah penting untuk mendapatkan rawatan perubatan sebaik sahaja anak saya menunjukkan tanda-tanda jangkitan pernafasan. (*I think it's critical to get medical attention as soon as my child exhibits signs of a respiratory infection*.)

☐  Sangat setuju (Strongly agree) ☐  Setuju (Agree) ☐  Tidak setuju (Disagree) ☐  Sangat tidak setuju (Strongly disagree) ☐  Tidak pasti (Not sure)

5.      Mengenai kesihatan pernafasan anak saya, saya percaya bahawa saya mempunyai sokongan perubatan yang mencukupi. (I*n regards to my child's respiratory health, I believe that I have enough medical support*.*)*

☐  Sangat setuju (Strongly agree) ☐  Setuju (Agree) ☐  Tidak setuju (Disagree) ☐  Sangat tidak setuju (Strongly disagree) ☐  Tidak pasti (Not sure)

6.      Saya berpendapat bahawa para profesional perubatan tidak melakukan cukup untuk membantu saya menguruskan keadaan pernafasan anak saya. (*I think that medical professionals are not doing enough to help me manage my child's respiratory condition*.)

☐  Sangat setuju (Strongly agree) ☐  Setuju (Agree) ☐  Tidak setuju (Disagree) ☐  Sangat tidak setuju (Strongly disagree) ☐  Tidak pasti (Not sure)

7.      Saya percaya bahawa lebih banyak nasihat tentang pengurusan kesihatan pernafasan harus diberikan oleh pasukan perubatan anak saya.(I* believe that more advice on managing respiratory health should be given by my child's medical team*.)

☐  Sangat setuju (Strongly agree) ☐  Setuju (Agree) ☐  Tidak setuju (Disagree) ☐  Sangat tidak setuju (Strongly disagree) ☐  Tidak pasti (Not sure)

Practice domain

1.      Saya sentiasa memerhatikan tanda-tanda masalah pernafasan pada anak saya. (*I keep an eye out for signs of respiratory illnesses in my child.*)

☐  Sangat setuju (Strongly agree) ☐  Setuju (Agree) ☐  Tidak setuju (Disagree) ☐  Sangat tidak setuju (Strongly disagree) ☐  Tidak pasti (Not sure)

2.      Setiap kali anak saya menunjukkan gejala masalah pernafasan, saya akan pergi ke klinik untuk mendapatkan nasihat perubatan. (*Whenever my child exhibits symptoms of respiratory distress, I will go to the clinic to get medical advice*.)

☐  Sangat setuju (Strongly agree) ☐  Setuju (Agree) ☐  Tidak setuju (Disagree) ☐  Sangat tidak setuju (Strongly disagree) ☐  Tidak pasti (Not sure)

3.      Untuk mengurangkan risiko jangkitan pernafasan anak saya, saya menggunakan langkah-langkah pencegahan (*seperti imunisasi dan menjaga kebersihan tangan). To lower my child's risk of respiratory infections, I use preventative measures (such as immunisations and hand cleanliness*).

☐  Sangat setuju (Strongly agree) ☐  Setuju (Agree) ☐  Tidak setuju (Disagree) ☐  Sangat tidak setuju (Strongly disagree) ☐  Tidak pasti (Not sure)

4.      Saya merawat jangkitan pernafasan pada anak saya yang mengalami palsi serebral mengikut arahan perubatan yang disyorkan. (*I treat respiratory infections in my child with cerebral palsy according to the suggested medical instructions*.)

☐  Sangat setuju (Strongly agree) ☐  Setuju (Agree) ☐  Tidak setuju (Disagree) ☐  Sangat tidak setuju (Strongly disagree) ☐  Tidak pasti (Not sure)

5.      Saya telah mengambil bahagian dalam inisiatif pendidikan tentang pengurusan kesihatan pernafasan kanak-kanak yang mengalami palsi serebral. (*I've taken part in education initiatives on managing the respiratory health of children with cerebral palsy*.)

☐  Sangat setuju (Strongly agree) ☐  Setuju (Agree) ☐  Tidak setuju (Disagree) ☐  Sangat tidak setuju (Strongly disagree) ☐  Tidak pasti (Not sure)

6.      Saya ingin menghadiri sesi latihan atau seminar tentang cara mengurus kesihatan pernafasan kanak-kanak yang mengalami palsi serebral. (*I want to go to training sessions or seminars on how to manage the respiratory health of children with cerebral palsy*.)

☐  Sangat setuju (Strongly agree) ☐  Setuju (Agree) ☐  Tidak setuju (Disagree) ☐  Sangat tidak setuju (Strongly disagree) ☐  Tidak pasti (Not sure)

7.     Ibu bapa kepada kanak-kanak yang mengalami palsi serebral harus mempunyai akses kepada lebih banyak bahan tentang pendidikan kesihatan pernafasan. (*Parents of children with cerebral palsy should have access to more respiratory health education materials.*)

☐  Sangat setuju (Strongly agree) ☐  Setuju (Agree) ☐  Tidak setuju (Disagree) ☐  Sangat tidak setuju (Strongly disagree) ☐  Tidak pasti (Not sure)

8.      Maklumat kesihatan pernafasan yang dibentangkan oleh pakar perubatan adalah sangat berguna. (*The respiratory health information that medical experts present is very useful*.)

☐  Sangat setuju (Strongly agree) ☐  Setuju (Agree) ☐  Tidak setuju (Disagree) ☐  Sangat tidak setuju (Strongly disagree) ☐  Tidak pasti (Not sure)
